# Supplementary material for: Content-rich biological network constructed by mining PubMed abstracts
Source: BMC Bioinformatics. 2004 Oct 8;5:147. doi: 10.1186/1471-2105-5-147 (PMC528731; doi:10.1186/1471-2105-5-147)
Supplement: Additional File 2 — The original results of the above study (non-essential files are deleted to keep the file size under the limit set by BMC bioinformatics). [file 1471-2105-5-147-S2.bz2 › chilibotAdditionalFile2/dip10/291ID1372994E1122/html/WEE1_CDC2.html]

 


 **WEE1** and **CDC2** 
  
Found 181 abstracts in PubMed, retrieved 10.  
 

 What does Google say? 
 PDF only 
| .edu only 

---

**Interactive relationship** (e.g. stimulation, inhibition, etc)

**Inhibitory relationship**- **Cdc2**  regulation also involves positive feedback, with active  **Cdc2**  cyclin B stimulating its activator Cdc25 refs 5 7 and inactivating its inhibitors  **Wee1**  and Myt1 refs 8 11.  Ref: 12629549 Nat Cell Biol, 2003
- The activity of  **cdc2**  associated kinase was decreased in both cell lines, which was accompanied by induction of  **Wee1** .  Ref: 12406077 Br J Haematol, 2002
- In particular, augmentation of CDK  [ **CDC2** ]  inhibitors p21cip1 and p27kip1 and  **Wee1**  kinase and diminution of CDC25C phosphatase coincided with induced Tyr 15 phosphorylation and inhibition of  **CDC2** .  Ref: 12669309 Mol Carcinog, 2003
**Neutral relationship**- We show that in G1 arrested orp1 4 cells,  **Wee1**  phosphorylates and inactivates  **Cdc2** .  Ref: 12186947 J Cell Sci, 2002
- Protein abundance for total  **Cdc2** ,  **Cdc2**  phosphorylated at the threonine 161 residue, Wee 1  [ **WEE1** ] , Myt 1, Chk 1, and 14 3 3sigma was not affected by treatment with ACNU in either cell line.  Ref: 12222840 J Neurooncol, 2002
- These data suggest that  **Wee1**  functions in normal postmitotic neurons, but is altered in AD so as to promote activation of  **Cdc2**  cyclin B1.  Ref: 12214061 J Alzheimers Dis< MedlineTA>J Alzheimers Dis, 2002
- Degradation of Tome 1 during G1 allows for wee 1  [ **WEE1** ]  accumulation during interphase, thereby providing a critical link between the APC and SCF pathways in regulation of cdk1  [ **CDC2** ]  cyclin B activity and thus mitotic entry and exit.  Ref: 12679038 Cell, 2003
- The Wee  [ **WEE1** ]  kinases block entry into mitosis by phosphorylating and inhibiting the activity of the mitotic cyclin dependent kinase, Cdk1  [ **CDC2** ] .  Ref: 12217326 Dev Biol, 2002

**Non-interactive relationship** (e.g. studied together, co-existance, homology, etc.)

- Although the levels of  **Wee1**  and  **Cdc2**  protein were increased after exposure to either beam,  **Wee1**  protein levels were influenced more by carbon ion beam irradiation than by gamma rays.  Ref: 12787730 Cancer Detect Prev, 2003
- These findings suggest that the G2 arrest produced by heavy ion beams, such as the carbon ion irradiation used in this study, might be associated with the overexpression of the  **Wee1**  protein and of  **Cdc2**  phosphorylation regulated by  **Wee1** .  Ref: 12787730 Cancer Detect Prev, 2003
- In cycling cells the timing of  **cdc2**  activation and onset of mitosis are determined by the  **Wee1**  tyrosine kinase.  Ref: 12214061 J Alzheimers Dis< MedlineTA>J Alzheimers Dis, 2003
- The enzymatic activities of cyclins D1, E, A, Cdk1  [ **CDC2** ] , Cdk4, Cdk6, Cdk7, and  **Wee1**  were measured using in vitro kinase assays.  Ref: 12601350 Hepatology, 2003
- These findings suggest that TGF beta 1 induced G2 arrest in Hep3B at least in part through stabilization of  **Wee1**  and subsequent increase in Tyr 15 phosphorylation and inhibition of  **CDC2** .  Ref: 12669309 Mol Carcinog, 2003
- Tome 1 associates with Skp 1 and is required for degradation of the cdk1  [ **CDC2** ]  inhibitory tyrosine kinase  **wee1** .  Ref: 12679038 Cell, 2003
- In this study, we investigated the expression of  **Wee1**  and  **Cdc2**  protein levels in order to analyze the G2 arrest caused by carbon ion beam irradiation.  Ref: 12787730 Cancer Detect Prev, 2003
